# The leptin to adiponectin ratio (LAR) is reduced by sleeve gastrectomy in adults with severe obesity: a prospective cohort study

**DOI:** 10.1038/s41598-020-73520-3

**Published:** 2020-10-01

**Authors:** M. F. Rafey, C. E. H. Fang, I. Ioana, H. Griffin, M. Hynes, T. O’Brien, O. McAnena, P. O’Shea, C. Collins, C. Davenport, F. M. Finucane

**Affiliations:** 1 Bariatric Medicine Service, Centre of Diabetes, Endocrinology and Metabolism, Galway University Hospitals and HRB Clinical Research Facility, National University of Ireland, Galway, Ireland; 2 Department of Medicine, National University of Ireland Galway, Galway, Ireland; 3 Department of Upper Gastrointestinal Surgery, National University of Ireland Galway, Galway, Ireland

**Keywords:** Predictive markers, Obesity

## Abstract

Bariatric surgery is known to reduce leptin and increase adiponectin levels, but the influence of sleeve gastrectomy on the leptin: adiponectin ratio (LAR), a measure of insulin sensitivity and cardiovascular risk, has not previously been described. We sought to determine the influence of sleeve gastrectomy on LAR in adults with severe obesity.In a single centre prospective cohort study of adults undergoing laparoscopic sleeve gastrectomy over a four-month period in our unit, we measured LAR preoperatively and 12 months after surgery. Of 22 patients undergoing sleeve gastrectomy, 17 (12 females, 12 with type 2 diabetes) had follow-up LAR measured at 12.1 ± 1 months. Mean body weight decreased from 130.6 ± 30.8 kg to 97.6 ± 21.6 kg, body mass index (BMI) from 46.9 ± 7.8 to 35.3 ± 7.2 kg m^−2^ and excess body weight from 87.5 ± 31.3 to 41.3 ± 28.8% (all *p* < 0.001). The reduction in leptin from 40.7 ± 24.9 to 30.9 ± 30.5 ng/ml was not significant (*p* = 0.11), but adiponectin increased from 4.49 ± 1.6 to 8.93 ± 6.36 µg/ml (*p* = 0.005) and LAR decreased from 8.89 ± 4.8 to 5.26 ± 6.52 ng/µg (*p* = 0.001), equivalent to a 70.9% increase in insulin sensitivity. The correlation with the amount of weight lost was stronger for LAR than it was for leptin or adiponectin alone. In this single-centre, interventional prospective cohort, patients undergoing laparoscopic sleeve gastrectomy had a substantial reduction in their LAR after 12 months which was proportional to the amount of weight lost. This may indicate an improvement in insulin sensitivity and a reduction in cardiovascular risk.

## Introduction

Obesity is a major determinant of type 2 diabetes risk, primarily through its adverse effects on insulin sensitivity^1^. Bariatric surgery to treat severe obesity leads to significant improvements in diabetes control^2^, cardiovascular risk^3,4^, microalbuminuria^5^ and systemic inflammation^6^. However, the influence of bariatric surgery on insulin sensitivity is not well defined^7,8^, partly because quantifying insulin sensitivity can be challenging. For example, the hyperinsulinaemic-euglycaemic clamp allows precise measurement of insulin sensitivity in muscle and liver^9^ but is a technically elaborate and expensive technique that is only available in specialized research units. The leptin: adiponectin ratio (LAR) is a measure of whole body insulin sensitivity that has been validated in large population-based studies^10,11^. Both of these hormones are adipokines, produced by adipocytes and they influence metabolic homeostasis. Leptin works through receptors in the hypothalamus to regulate dietary intake as well as energy expenditure^12^ and has been shown to be elevated in individuals with obesity^13^. Conversely, adiponectin reduces circulating free fatty acids by increasing tissue fat oxidation: Adiponectin levels tend to be lower in individuals with obesity^14^. Several studies have described reductions in leptin and increases in adiponectin in adults with severe obesity undergoing bariatric surgery^3,6–8,15^. Recently, investigators described changes in the adiponectin: leptin ratio (ALR) in a cohort of 25 Spanish adults with type 2 diabetes who underwent Roux-en-Y gastric bypass^16^. To date, the influence of sleeve gastrectomy on the ratio of these two hormones has not been described. We sought to quantify the effects of sleeve gastrectomy on leptin, adiponectin and LAR in adults with severe obesity, and to assess whether the magnitude of weight loss after surgery influenced the change in LAR.

## Methods

We conducted a single-centre, interventional prospective cohort study of all patients undergoing laparoscopic sleeve gastrectomy at our hospital over four months between September and December 2016. We performed follow-up measures 12.1 ± 1 months after surgery. All study participants provided written informed consent and the study was approved by the Galway University Hospital’s Research Ethics Committee (reference C.A. 2058). The study was conducted adhering to the STROBE (Strengthening the Reporting of OBservational studies in Epidemiology) guidelines^17^.

Inclusion criteria for bariatric surgery at our institution are consistent with those internationally. We define severe obesity as a body mass index (BMI) ≥ 40 kgm^−2^ (or ≥ 35 kgm^−2^ with co-morbidities such as type 2 diabetes and obstructive sleep apnea syndrome). Male and female patients aged 18 years or older, put forward for consideration for sleeve gastrectomy by the bariatric multidisciplinary team at our institution (nurse, dietitian, physician, psychologist, surgeon) were all eligible for inclusion. Our clinical practice is to refer these patients for surgical consideration after completion of a ten-week structured lifestyle modification programme that we have described in detail previously^18^. Patients must have undertaken a formal psychological assessment of the suitability of sleeve gastrectomy for their treatment. Those with a recent myocardial infarction (within six months), untreated arrhythmia, untreated left ventricular failure, recent cholelithiasis (within the past year), type 1 diabetes, untreated major psychiatric disorders, eating disorders, undergoing cancer treatment, or a BMI < 35 kg m^−2^ or those deemed unlikely to attend for post-operative follow-up (e.g. frequent clinic non-attendance) were excluded from undergoing sleeve gastrectomy. The study population was a convenience sample and its size was determined by the number of sleeve gastrectomies done during the study period. All metabolic and anthropometric baseline and follow-up measures were conducted at our bariatric out-patient clinic.

Weight was measured on a Tanita® scale and height with a Seca® wall-mounted stadiometer. Blood pressure was measured with an automated oscillometric device (Omron®) using a large cuff on the right arm, after participants had been seated quietly for five minutes. Three measures were recorded at one-minute intervals and the average of the three was recorded. Blood samples were drawn from patients in the fasted state on the morning of their sleeve gastrectomy. Glycosylated haemoglobin (HbA1c) was measured using High Performance Liquid Chromatography (HPLC) on the Menarini® HA8160 analyser. Leptin and adiponectin were measured using separate two-site micro titre plate-based DELFIA assays manufactured by R&D Systems Europe, Abingdon UK. The adiponectin assay measured “total” adiponectin and our “in-house” analyses have found a between batch imprecision of 5.4% at 3.6 µg/ml, 5.2% at 9.2 µg/ml and 5.8% at 15.5 µg/ml, as previously described^19^. For leptin, the between batch imprecision was 7.1% at 2.7 ng/ml, 3.9% at 14.9 ng/ml and 5.7% at 54.9 ng/ml.

All statistical analyses were conducted with SPSS® version 24. Changes in anthropometric and metabolic variables between baseline and follow-up were assessed using the student’s paired t-test, assuming equal variances. The relationship between the degree of weight loss over 12 months and changes in LAR was determined using Pearson correlation and linear regression modelling.

### Human and animal rights

This study was conducted according to the principles established in the Declaration of Helsinki and described by the International Conference for the Harmonisation of Good Clinical Practice.

### Informed consent

Written informed consent was obtained from all individual participants included in the study.

## Results

Of 148 patients with severe obesity who were on the waiting list for sleeve gastrectomy at the time the study started, 22 were put forward for surgery (based on clinical prioritisation and length of time waiting, under a short-term, government-funded “waiting list initiative”). All of these patients were invited to the study and all agreed to participate. However, one patient subsequently died six months after sleeve gastrectomy, from an aggressive oesophageal carcinoma with liver metastases which first presented three months after his sleeve gastrectomy. This was despite the patient having a normal barium swallow and ultrasound before his bariatric surgery. Four patients attended for bariatric follow-up at other institutions and were unable to attend within our specified window of 10–14 months post-surgery for follow-up measures. Thus, we report on 17 patients for whom baseline and 12-month follow-up measures were available. 12 patients were female and 12 had type 2 diabetes at baseline. The mean duration of diabetes was 9.9 (range 3–27) years. Mean age was 52.2 ± 8.3 (range 39–71) years, with a follow- up interval of 12.1 ± 1 (range 10–13) months. Baseline and follow-up anthropometric and metabolic characteristics are presented in Table 1. There were substantial reductions in weight, BMI and excess body weight over 12.1 months, as shown, with a mean weight loss of 33.0 ± 21.6 kg and an absolute reduction in excess body weight percentage (EBW%) of 47.2 ± 28.8% (all *p* < 0.001), equivalent to a percentage total weight loss of 24.3 ± 12%.

**Table 1:** Baseline and follow-up anthropometric and metabolic characteristics in 17 patients, 12 months after sleeve gastrectomy.

Variable	N	Baseline		Follow-up		*p* value
		Mean	± SD	Mean	± SD	
Weight (kg)	17	130.6	± 30.8	97.6	± 21.6	< 0.001
BMI (kg/m^2^)	17	46.9	± 7.8	35.3	± 7.2	< 0.001
EBW (%)	17	87.5	± 31.3	41.3	± 28.8	< 0.001
SBP (mmHg)	17	124.4	± 13.0	126.8	± 18.4	0.067
DBP (mmHg)	17	72.2	± 10.9	71.3	± 12.5	0.83
HbA1c (mmol/mol)*	12	62	± 13.2	53.3	± 11.8	0.16
Leptin (ng/ml)	17	40.7	± 24.9	30.9	± 30.5	0.11
Adiponectin (µg/ml)	17	4.49	± 1.6	8.93	± 6.36	0.005
LAR (ng/µg)^#^	17	7.16	[5.21, 10.59]	2.59	[1.14, 7.44]	0.02
LAR (ng/µg)^#^*	12	8.54	[5.09, 10.37]	4.2	[2.24, 7.56]	0.006

*Denotes subgroup of patients with type 2 diabetes at baseline.

Data are presented as mean ± standard deviation (or # median and interquartile range for LAR, which was not normally distributed).

All variables were compared using the paired t-test, except for LAR which was compared using the Wilcoxon Signed Rank Test.

*BMI* body mass index, *EBW* excess body weight, *SBP* systolic blood pressure, *DBP* diastolic blood pressure, *LAR* leptin: adiponectin ratio.

There was a non-significant trend to reduced HbA_1c_ overall after 12 months, which was more pronounced (though remained non-significant) in the subgroup of patients with diabetes. Two patients were taking insulin preoperatively and both remained on insulin 12 months later. Of two patients taking sodium glucose linked transported 2 (SGLT2) inhibitor therapy preoperatively, one remained on it and a second had stopped it. A third patient had started the drug by the time of their follow-up. Of 12 patients taking metformin at baseline, three had stopped this by 12 months. Of five patients taking glucagon like peptide 1 (GLP1) agonist therapy at baseline, one remained on this at follow-up. All three patients on gliptin therapy for diabetes had this stopped at the time of their surgery, while no patients were taking sulphonylureas or glitazones at baseline or follow-up. Ten patients were taking statin therapy at baseline and this was continued in all patients routinely post-operatively. There was a reduction in the prevalence of patients taking antihypertensive therapy from 71 to 29% over 12 months.

Overall, there was a (non-significant) trend to reduced leptin levels, while there was a more pronounced and statistically significant increase in adiponectin. This equated to an overall reduction in the LAR which was consistent with a 70.9% increase in insulin sensitivity in the cohort 12 months after sleeve gastrectomy. When examining changes in the subgroup of patients with diabetes, results were similar to the group overall, as shown.

**Figure 1:**
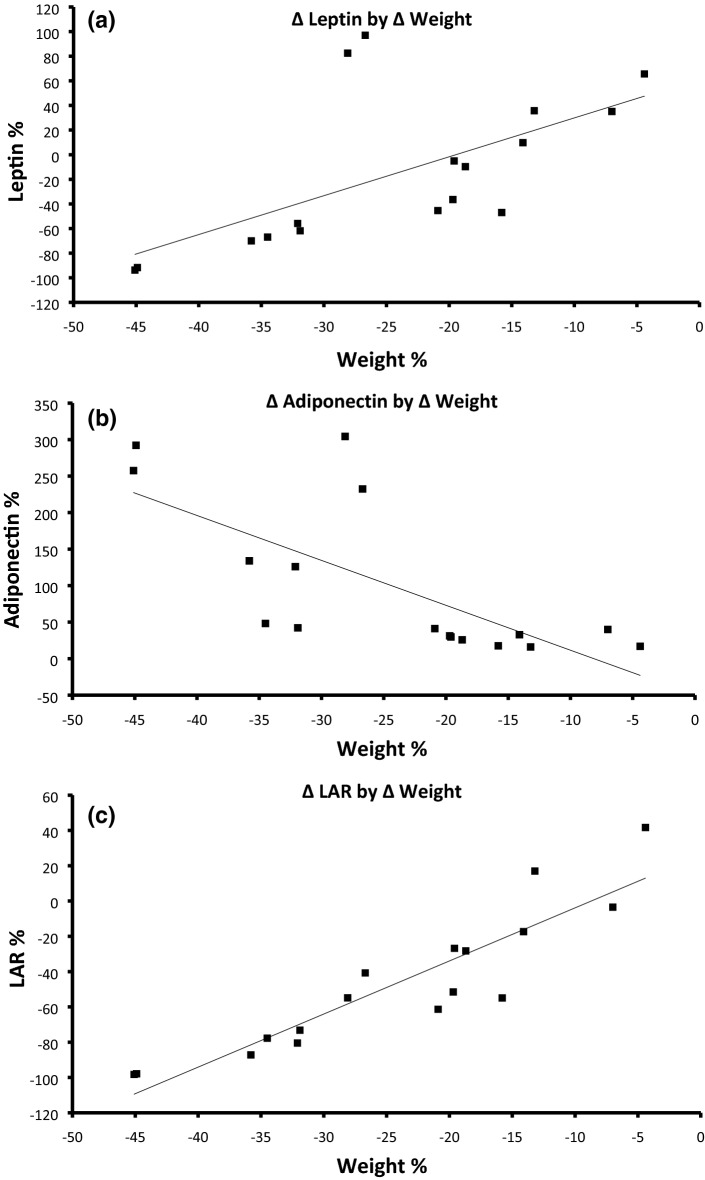
Correlations betsssween change in percentage body weight and relative changes in leptin, adiponectin and LAR.

Next, we sought to determine the correlation between the percentage weight lost and changes in leptin, adiponectin and the LAR. As outlined in Fig. 1a–c, there was a modest correlation only between the percentage weight lost with leptin and with adiponectin individually, such that the percentage weight loss accounted for approximately 39.5% of the reduction in leptin (r^2^ = 0.3948, *p* = 0.007) and 49.6% of the increase in adiponectin (r^2^ = 0.4961, *p* = 0.002), respectively. However, there was a much stronger correlation seen for the LAR, such that approximately 82.2% of the reduction in LAR was accounted for by the percentage weight lost (r^2^ = 0.8222, *p* < 0.001).

## Discussion

We have shown that in a predominantly white cohort of adults with severe obesity who underwent laparoscopic sleeve gastrectomy, there was a substantial reduction in the LAR after 12 months, which may indicate an increase in insulin sensitivity. While several studies have measured leptin or adiponectin separately in patients after bariatric surgery^3,6–8,15^, we are aware of only one other study by Unamuno et al. that has considered them together as a ratio and as an indicator of adipocyte dysfunction^16^. Ours is the first study to consider these adipokines as a ratio in patients undergoing laparoscopic sleeve gastrectomy. We found that the reductions in LAR were driven primarily by an increase in adiponectin rather than a reduction in leptin, and these changes were proportional to the reduction in weight after surgery, consistent with the Unamuno study. The changes we observed in leptin and adiponectin concentrations were less than those described after bypass bariatric surgery^3,6,7^ and are more consistent with those previously described after banding^8^.

We did not see a statistically significant reduction in leptin, but we think this is due to inadequate power, given that a recent randomised controlled trial confirmed that leptin decreases after sleeve gastrectomy^20^. As the primary effect of leptin is to supress appetite and defend against weight gain via hypothalamic signalling, it might seem counterintuitive to find a reduction rather than an increase after sleeve gastrectomy. However this phenomenon is well described and may be due to ‘leptin resistance’, whereby individuals with obesity demonstrate paradoxically high levels of circulating leptin but diminished leptin sensitivity^21^. Previous studies have suggested that decreased leptin levels after bariatric surgery do not attenuate weight loss, because of compensatory increased leptin sensitivity in the hypothalamus^22^. The mechanisms by which leptin sensitivity might be restored remain to be determined, but other adipokines such as fibroblast growth factor-21 (FGF-21) may mediate this effect via direct signalling in the central nervous system or through augmenting the secretion of adiponectin^23^. Another consideration is the interaction between leptin and adiponectin. For example, in normal-weight individuals, leptin enhances adiponectin secretion, but this effect is lost in patients with obesity through the action of caveolin-1, which attenuates leptin-dependant increases in adiponectin^24^.

Increased circulating levels of adiponectin are not always indicative of improvements in health or reductions in cardiovascular risk: the so-called “adiponectin paradox” refers to the observation that while adiponectin mediates a variety of essentially beneficial effects and levels of adiponectin correlate positively with better long term cardiovascular outcomes in young healthy populations they also correlate with increased risk of premature death in high-risk populations, especially older patients or those with ischemic heart disease, heart failure or renal failure^25^.

Of note, the mean HbA_1c_ was low at baseline in our patients with diabetes. This is likely due to relatively short-term improvements in glycaemic control that occur with our pre-operative “high protein diet” that we use in order to reduce the size of the liver. Data regarding HbA1c in the period preceding surgery are not to hand. However, we don’t think that this would affect our findings as it is likely that the pre-operative diet would have led to increased insulin sensitivity, reflected in a reduced LAR at baseline, thus if anything attenuating our ability to detect a change in LAR after surgery. Future studies could incorporate measurement of HbA1c, leptin, adiponectin and LAR just before the pre-operative high protein diet commences, in order to determine the extent to which these variables change before surgery.

A key limitation of our study is its modest size (reflected in the borderline statistical significance of the reduction in LAR, for example), but the effects of bariatric surgery on metabolic outcomes and cardiovascular disease risk tend to be so profound that even in randomised controlled trials, surgical interventions require population sizes^2^ that are orders of magnitude smaller than those in drug^26^ or lifestyle intervention trials^27^. Our cohort size is similar to other surgical studies^3,6–8,15^ and our findings around the correlation between weight loss and reduced LAR are statistically robust. Rather than using LAR as the only index of insulin sensitivity, future studies could also examine indices related to fasting glucose and insulin (such as the homeostasis model assessment, HOMA^28^), in order to strengthen causal inference on the impact of bariatric interventions on insulin sensitivity.

There were changes in diabetes medication usage within the cohort that are unavoidable in an observational study such as this, which might have influenced our results. For example, metformin is known to have a weak positive effect on insulin sensitivity^29^ and potentially a lowering effect on circulating levels of leptin^30^, so it is possible that the cessation of metformin diminished the reduction in the LAR observed in our patients. Also, GLP agonist therapy has previously been reported to attenuate the reduction in circulating levels of leptin after weight loss^31^, so the cessation of GLP agonist therapy in four of our patients may have diminished the reduction in leptin that we observed. Similar considerations apply to the three patients who stopped gliptin medications, as these drugs decrease leptin and increase adiponectin^32^. SGLT2 inhibitors decrease leptin and increase adiponectin^33^, so we do not think that initiation of this treatment in one patient and its cessation in another is likely to have led to the observed difference in LAR after the programme. Angiotensin converting enzyme (ACE) inhibitors increase adiponectin^34^, so we think their cessation would have attenuated rather than enhanced the observed reduction in LAR. Thus, we would expect that the overall reductions in the usage of these drugs would have led to attenuation, rather than exaggeration of the measured differences in adipokines and LAR that we observed.

Ultimately, this work could inform definitive interventions and aetiological trials in larger populations of patients to overcome these limitations and determine the efficacy of sleeve gastrectomy and other interventions to reduce insulin resistance. Whether LAR is a good way to identify “responders” to surgery remains to be determined in large, prospective studies.

## Conclusions

This single-centre, interventional prospective cohort study of adults with severe and complicated obesity undergoing laparoscopic sleeve gastrectomy found that after twelve months there was a substantial reduction in the leptin: adiponectin ratio and that this reduction was proportional to the amount of weight lost after surgery. Given the heterogenous nature and small size of the study population, the findings must be regarded as preliminary. Nonetheless they suggest a change in adipocyte function consistent with improved insulin sensitivity. Further studies in larger, more specifically defined patient subgroups would help to further elucidate the relevance of adipokine measurement in these patients and the mechanistic basis for metabolic improvements after bariatric surgery.
